# The Complete Mitochondrial Genome and Phylogenetic Analysis of the Freshwater Shellfish *Novaculina chinensis* (Bivalvia: Pharidae)

**DOI:** 10.3390/ijms25010067

**Published:** 2023-12-20

**Authors:** Ziquan Zhou, Yuxin Song, Zewen Zheng, Yunguang Liu, Haiyan Yao, Xiaozhen Rao, Gang Lin

**Affiliations:** Fujian Key Laboratory of Special Marine Bioresource Sustainable Utilization, College of Life Sciences, Fujian Normal University, Fuzhou 350117, China; weatherzzq@163.com (Z.Z.); elaine9907@163.com (Y.S.); zhengzewen2019@163.com (Z.Z.); 15060110810@163.com (Y.L.); daphneyanng@163.com (H.Y.)

**Keywords:** razor clams, Pharidae, *Novaculina chinensis*, mitogenome, phylogenetic relationship

## Abstract

Razor clams, belonging to the Pharidae and Solenidae families, are ecologically and economically important; however, very little research has been conducted on the Pharidae family. The genus *Novaculina* is a marine-derived freshwater lineage, and *Novaculina chinensis* is a rare freshwater species of the Pharidae family. In order to understand the phylogenetic relationships of *N. chinensis*, we sequenced the mitochondrial genome of the genus *Novaculina*, which is 16,262 bp in length and consists of 12 protein-coding genes (PCGs), 22 transfer RNA genes (tRNAs), and 2 ribosomal RNA genes (rRNAs). The phylogenetic relationships of 69 Imparidentian mitochondrial genomes (mitogenomes) indicated that *N. chineisis* is closely related to *Sinonovacula constricta* of the order Adapedonta. Our study also found that the Ka/Ks ratios of 12 protein-coding genes in the Pharidae family are lower than one, indicating the occurrence of negative purification selection. Morphological observations of the siphons of *N. chinensis*, *Novaculina myanmarensis*, and *Novaculina gangetica* indicate that *N. chinensis* may be the ancestral clade of the genus *Novaculina*, which has not been proposed in previous studies. Our study provides useful molecular information on the phylogenetic and evolutionary relationships of Pharidae and also contributes to the conservation and management of the germplasm resources of *N. chinensis*.

## 1. Introduction

**Figure 1:**
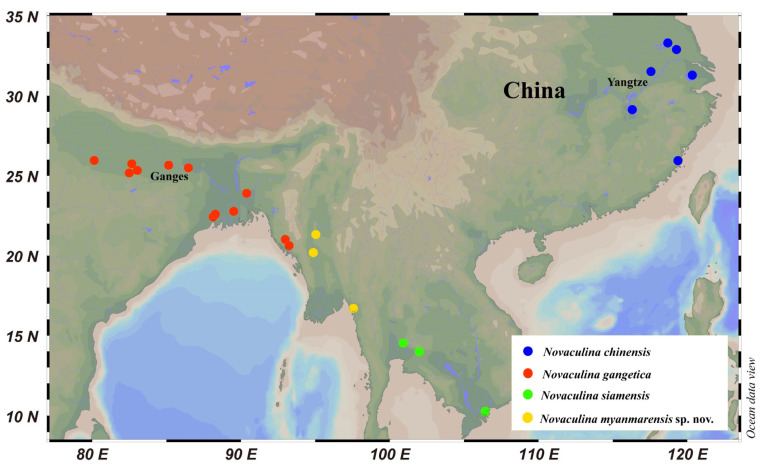
Distribution range of the genus *Novaculina* based on those references [3,4,5,6].

Bivalves have a wide range of taxa in marine, brackish, and freshwater. Razor clams are important deep-burrowing bivalves, most of which are found in the shallow waters of the tropical, subtropical, and temperate seas, and belong to two families (Pharidae and Solenidae) in the superorder Imparidentia [1,2]. Although Pharidae is mainly composed of marine species, it includes one genus that represents four freshwater species that are geographically separated from each other [3]. These four species are distributed throughout various freshwater drainages in Asia, from the Ganges River located in India to the Yangtze River situated in China (Figure 1) [4]. 

*Novaculina chinensis* was first reported in Taihu and Gaoyou Lake, Jiangsu Province, China [5]. It is mainly distributed in the middle and lower reaches of the Yangtze River in China and the Minjiang River in Fuzhou, China [6]. Due to overfishing, water pollution, and habitat changes, the population of *N. chinensis* has declined significantly in all regions and its germplasm resources are in danger of being depleted [7]. Previous studies of *N. chinensis* have mainly focused on its reproductive cycle, morphology, nutritive compositions, and the ultrastructure of its sex gonads [7,8,9,10,11]. The research on the genus *Novaculina* located in Southeast Asia has mainly focused on its biodiversity and biogeography [3,4]. Prior to this work, there was little research at the molecular level for this genus. Only the *cox1*, *16S* rRNA (*rrnL*), and *28S* rRNA (*rrnS*) genes of *N. gangetica* and *N. myanmarensis* were sequenced. 

The superorder Imparidentia is a newly defined branch of bivalves encompassing diverse clades of marine, brackish, and freshwater bivalve mollusks [12]. It includes five major lineages: Lucinida, Cardiida, Adapedonta, Myida, and Venerida [2]. However, the phylogenetic relationship of *N. chinensis* in Imparidentia is not clear due to it not being analyzed before. The mitochondrial genome (mitogenome) of classic metazoans is a small and compact circular molecule that typically includes 13 protein-coding genes (PCGs), 2 ribosomal RNA genes (rRNAs), and 22 transfer RNA genes (tRNAs), with the exception of bivalve shellfish in which loss of the *atp8* gene is observed in some species [13,14]. The complete mitochondrial genome has been widely used for studying the phylogenetic reconstruction and adaptative evolution due to its maternal inheritance, low intermolecular recombination, high copy number, and high replacement rate [2,15,16]. 

In this study, we sequenced and described the complete mitochondrial genome of *N. chinensis*, with the aim of analyzing the genomic features of its mitogenome, including the genome structure, nucleotide composition, and codon usage, as well as the selection pressure in the Pharidae family. Moreover, we constructed a phylogenetic tree to infer the phylogenetic position of *N. chinensis* in the superorder Imparidentia. Overall, our study provides useful molecular data to better understand the phylogenetic relationship and evolutionary journey of the genus *Novaculina*, which is important for the conservation and management of the shellfish germplasm resources.

## 2. Results and Discussion

### 2.1. Genome Features

**Figure 2:**
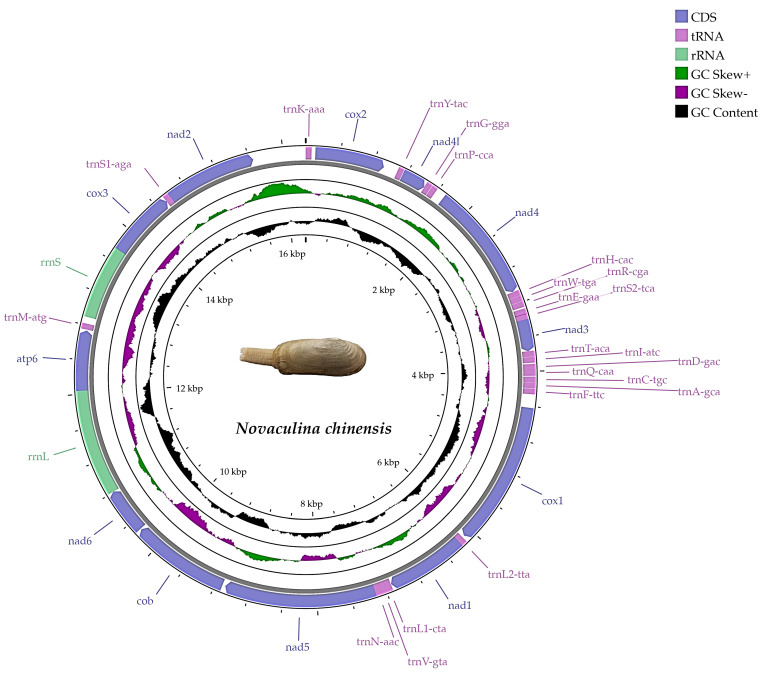
Mitogenome map of *N. chinensis*.

The complete mitogenome of *N. chinensis* is 16,262 bp in length (Figure 2). It has 12 PCGs (except *atp8*), 22 tRNA genes, and 2 rRNA genes. All of the genes were encoded on the heavy strand (Table 1). The base composition was as follows: A (28.15%), T (43.45%), G (18.86%), and C (9.54%). The A + T content (71.60%) was higher than the G + C content (28.40%), indicating a significant AT bias (Table 2). The AT skew value was −0.214, while the GC skew value was 0.328. Four overlaps were detected in the mitochondrial genome, and among these, the largest overlap was found between *trnE* and *trnS2*. The length of *N. chinensis* was smaller than other species in the Pharidae family, but the AT content was higher than other species in the Pharidae family (Table S1).

In the present study, the mitochondrial genome structure of *N. chinensis* was found to be largely consistent with that of other published species in the Pharidae family, showing a high degree of conservation. The mitogenomes of *Siliqua minima*, *Cultellus attenuatus*, *S. constricta*, and *N. chinensis* had the same genome organization, containing 12 PCGs (except *atp8*), 22 tRNAs, and 2 rRNAs, respectively; they have the same gene order without gene rearrangement and four mitogenomes varied in size from 16,262 bp to 17,225 bp [15,17,18]. The mitogenome of *N. chinensis* has a different mitochondrial genome size and nucleotide composition than other species in the Pharidae family. The small genome size and high AT content is a distinctive feature of *N. chinensis*. Wu et al. [19] and Annam et al. [20] sequenced the mitogenomes of five freshwater mussels in the Unionidae family; they had the same features of mitogenome including 13 PCGs, 22 tRNAs, 2 rRNAs, and one female specific gene (FORF). Among the 38 mitochondrial genes, 11 genes were encoded on the heavy chain and the remaining 27 genes were encoded on the light chain; however, all the mitochondrial genes of *N. chinensis* were encoded on the heavy strand, yet this phenomenon generally occurs in the mitochondrial genomes of marine bivalves [13,21]. Recently, multi-locus phylogenetic analyses have supported the genus *Novaculina* as a relict marine freshwater lineage [3,22]. Therefore, our findings have indicated that the mitogenome of *N. chinensis* retains some of the characteristics of marine bivalves.

### 2.2. Protein-Coding Genes, Transfer RNA, and Ribosomal RNA Genes

The mitogenome of *N. chinensis* has 12 PCGs and lacks the *atp8* gene. The total length of all 12 PCGs of *N. chinensis* is 11,502 bp, accounting for 70.73% of the complete length of the mitogenome (Table 2). For all 12 PCGs identified in the *N. chinensis* mitogenome, three genes (*cox1*, *nad5,* and *nad6*) were initiated with the start codon ATT, and the remaining nine genes had the start codon ATG. The *cox2*, *nad4*, *nad3*, *nad6,* and *cox3* genes carried the termination codon TAG (Table 1). Moreover, the most common termination codon TAA was detected in seven PCGs.

The absence of the *atp8* gene has been suggested for most bivalves, especially marine species with the exception the venerid *Venerupis philippinarum*, the hiatellid *Hiatella arctica*, and unionid species [15,23]. The absence of *atp8* is also observed in the mitogenomes of three species in the Pharidae family: *S. minima*, *C. attenuatus*, and *S. constricta* [15,17,18]. ATP synthase is the final enzymatic complex in the respiratory chain, directly producing ATP as it couples with the electrochemical gradient of the inner mitochondrial membrane [24,25]. Sun et al. [24] suggest that the *atp8* genes might be relaxed from selective constraints because of the change in locomotive ability and the reduced energy requirements that emerged in marine bivalve evolution. The lack of the *atp8* gene in deep-burrowing bivalves of the Pharidae family may be due to its weak motility and low energy requirements.

**Figure 3:**
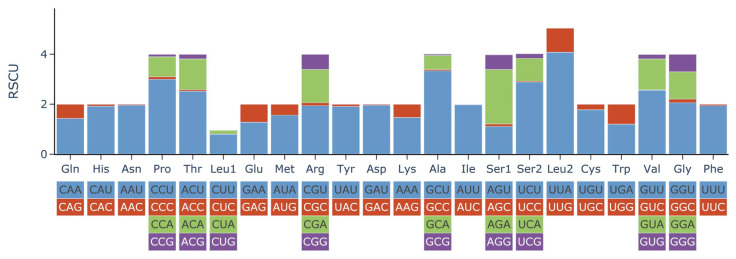
Relative synonymous codon usage (RSCU) of 12 PCGs in the mitogenome of *N. chinensis*.

Most invertebrates utilized two to four codons to encode its amino acids [26]. The nucleotide relative synonymous codon usages (RSCUs) of *N. chinensis* are presented (Figure 3, Table 3). UUA (Leu2), GCU (Ala), CCU (Pro), and UCU (Ser) are the most frequently used codons, whereas CUC, CUG (Leu1), AUC (Ile), and GUC (Val) are relatively scarce. As per the RSCU values, codons ending with an A or T were preferred. The two most frequent amino acids in the PCGs of *N. chinensis* were Ser and Leu.

Similar to most bivalves, the mitochondrial genome of *N. chinensis* contained 22 transfer RNA genes and 2 ribosomal RNA genes. The size of the 22 tRNA genes varied from 63 to 68 bp, and all of these genes can be classified into typical secondary structures (except two *trnS* genes). The two ribosomal RNA genes included *rrnL* and *rrnS*, with the former having a length of 1232 bp (between *nad6* and *atp6*), and the latter standing at 849 bp (between *trnM* and *cox3*). 

### 2.3. KaKs Analysis

The calculation of non-synonymous substitutions (Ka) and synonymous substitutions (Ks) is crucial for constructing a phylogenetic tree and understanding the evolutionary dynamics of protein-coding genes (PCGs) in closely related species [27,28]. The Ka/Ks ratio is used to determine whether selective pressure acted on PCGs during evolution: Ka/Ks > 1, positive selection; Ka/Ks = 1, neutral selection; and Ka/Ks < 1, negative selection [29,30]. 

To analyze the selection pressure on mitochondrial PCGs of the species in the Pharidae family, the Ka/Ks ratio of 12 PCGs were calculated. The Ka/Ks ratios of 12 PCGs are less than one (ranging from 0.0640 to 0.2311) (Table S2), indicating that those mitochondrial PCGs were under strong negative or purifying selection. The mitochondrial *cox1* gene had the smallest Ka/Ks value and bears the largest purifying selection pressure, while the highest Ka/Ks value is *nad6*. In most metazoans, the mitochondrial genes coding for the cytochrome c oxidase and cytochrome b are more conserved than the genes coding for NADH dehydrogenase [31]. Sun et al. [24] suggested that less motile bivalves survive and reproduce with lower metabolic efficiency, which may have accumulated more non-synonymous mutations in their mitochondrial genomes. The species in the Pharidae family have lower energy demands due to the fact that they are deep-burrowing bivalves. Therefore, we suggest that the species in the Pharidae family have also accumulated more non-synonymous mutations. However, there is relatively little useful molecular information in the Pharidae family and more research is required to understand the evolution of mitochondrial genes. 

### 2.4. Analysis of Phylogenetic 

**Figure 4:**
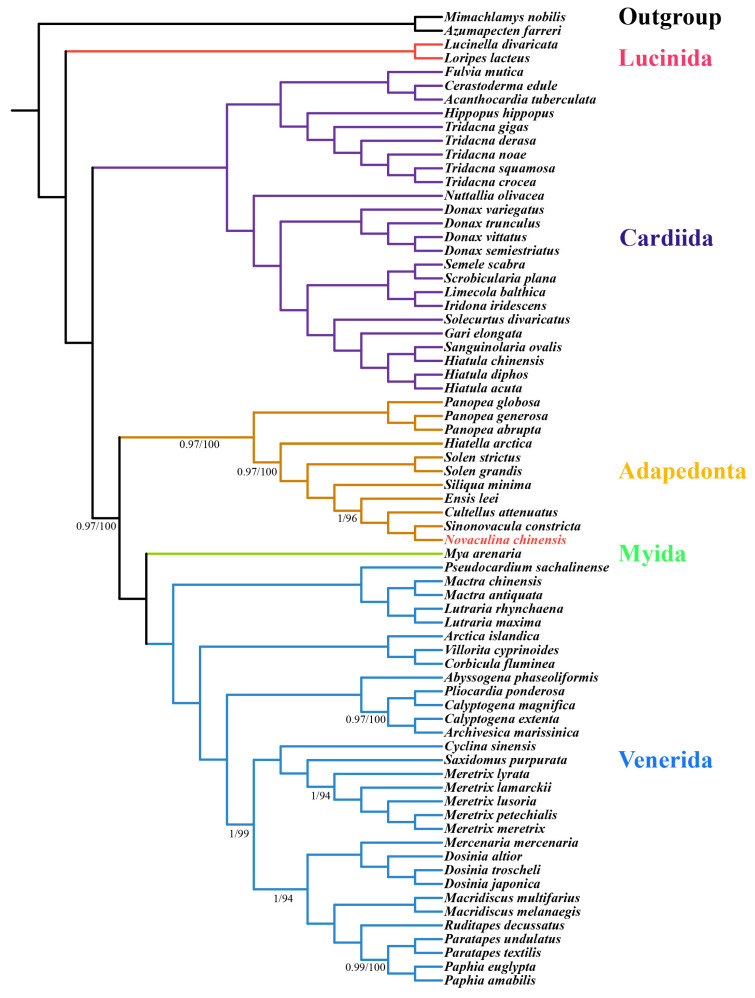
The phylogenetic tree for *N. chinensis* based on 12 PCGs. Below the nodes, the left is the Bayesian posterior probability (PP) value, and the right is the bootstrap proportion (BP) value. Nodes with no labels were maximally supported (PP/BP = 1/100). The red font is *N. chinensis*.

To explore the phylogenetic implications of the *N. chinensis* mitogenome in Imparidentia, we reconstructed a phylogenetic tree. Using Bayesian inference (BI) and maximum likelihood (ML) analyses of twelve protein-coding genes (except *atp8*) from sixty-nine species, we obtained nearly identical topologies with both methods (Figure 4), with high support in most nodes. Through the prism of phylogenetic analysis, we can observe the orders of Imparidentia, comprised Venerida, Cardiida, Adapedonta, and Lucinida, which is consistent with the phylogenetic trees constructed by Wang et al. [2] and Lemer et al. [32]; the latter of which utilized transcriptional data and morpho-anatomical features. Additionally, this result is largely consistent with the mitogenome-based phylogenetic tree constructed by Feng et al. [15]. Furthermore, the BI and ML analyses support the monophyly of the families Pharidae, Solenidae, and Hiatellidae, with each forming a distinct assemblage. Both analyses confirmed the sister group relationship of Pharidae and Solenidae, whereas Hiatellidae was identified as the sister to Pharidae and Solenidae. Notably, our findings indicate that *N. chinensis* and *S. constricta* in the Pharidae family constitute a new branch that is closely related to *C. ttenuates*, *E. leei*, and *S. minima* within the same family. The phylogenetic relationships between 11 species in the order Adapedonta are (*Panopea globose* + (*Panopea abrupta* + *Panopea generosa*)) + *Hiatella arctica* + (*Solen grandis* + *Solen strictus*) + (*S. minima* + *E. leei* + *C. attenuatus* + (*S. constricta* + *N. chinensis*)).

**Figure 5:**
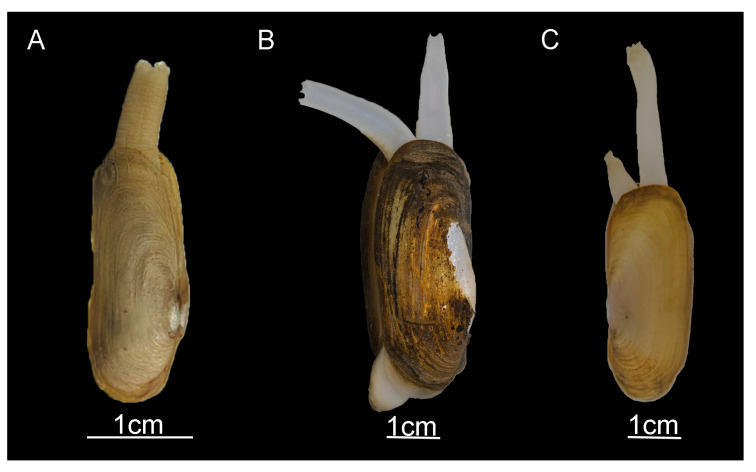
Three species of the genus *Novaculina*. (**A**) *N. chinensis*, (**B**) *N. gangetica*, and [3] (**C**) *N. myanmarensis* [3].

**Figure 6:**
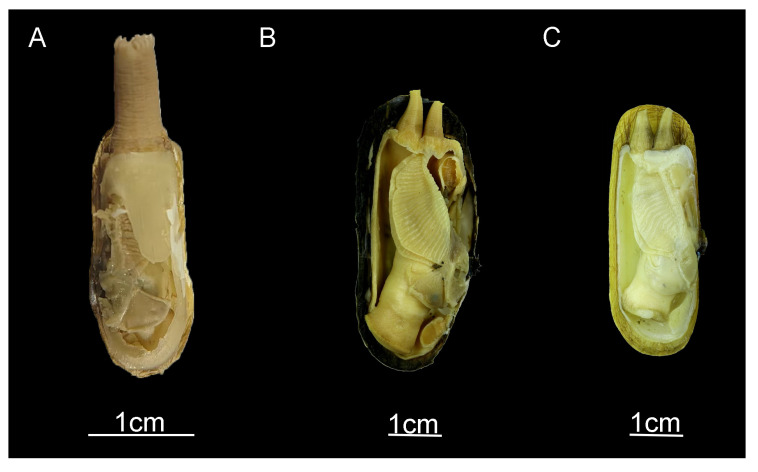
Soft body morphology of three species of the genus *Novaculina*. (**A**) *N. chinensis*, (**B**) *N. gangetica*, and [3] (**C**) *N. myanmarensis* [3].

Siphons play a crucial role in bivalve classification [33,34,35]. According to recent studies [3], we find that *N. chinensis* differs significantly in terms of morphology from *N. myanmarensis* and *N. gangetica*, which are distributed in Southeast Asia. Our observations indicate that *N. chinensis* has long, fused siphons, while the *N. myanmarensis* and *N. gangetica* siphons are long and separate (Figure 5 and Figure 6). We did not make comparisons with *N. siamensis* due to insufficient morphological information. Siphon size is one of the main factors determining the burying depth of benthic bivalves and thus plays a critical role in their survival [36]. *Novaculina chinensis*, *N. myanmarensis*, and *N. gangetica* all have long siphons, the only difference being whether the siphons are separated or not. Bivalves with long, separate siphons that remain active and extensive are better suited for survival than long, fused siphons because they can extend from the bivalve far above the surrounding surface to pick up food [37]. Long, fused siphons are more primitive from an evolutionary standpoint. Therefore, we hypothesize that *N. chinensis* may be the ancestral lineage of *N. myanmarensis*. and *N. gangetica*. However, to understand this process of evolution, additional molecular information on the genus *Novaculina* is necessary.

## 3. Materials and Methods

### 3.1. Sample Collection and DNA Extraction

The samples were collected from Taojiang River, Fuzhou city, Fujian Province. Morphological traits of the specimen were identified based on the following available sources from the literature [5,9]. Samples were photographed using a dissecting microscope. Total genomic DNA was extracted from the adductor muscle tissue using the DNeasy tissue kit (Qiagen, Beijing, China) and the procedure was conducted in accordance with the manufacturer’s protocols. The animal study protocol was approved by the Animal Ethics and Welfare Committee of Fujian Normal University (Approval No. IACUC-20230030).

### 3.2. Sequencing and Genome Assembly

The purified DNA was fragmented to ~500 bp using the Covaris M220 system, used to construct short-insert libraries according to the manufacturer’s instructions (TruSeqTM Nano DNA Sample Prep Kit, Illumina, Shanghai, China), and was then sequenced on an Illumina NovaSeq 6000 platform (BIOZERON Co., Ltd., Shanghai, China) with a 150 bp paired-end reads length, where raw reads were filtered using Trimmomatic 0.39 [38]. The mitogenome was generated via de novo assembly using GetOrganelle v1.7.5 as a reference for the mitochondrial genomes of closely related species (GeneBank No. EU880278.1 for *S. constricta* and MW653805.1 for *C. attenuatus*). 

### 3.3. Annotation and Sequence Analysis of Mitogenome

The mitochondrial genes were annotated using the online MITOS tool [39]. Default parameters were applied to predict protein-coding genes (PCGs), transfer RNA (tRNA) genes, and ribosomal RNA (rRNA) genes. The position of each coding gene was determined using BLAST searches against reference mitochondrial genes. Manual corrections of the start/stop codons of the genes were performed in SnapGene Viewer by referencing the reference mitogenomes. The circular mitogenome map of *N. chinensis* was drawn using Proksee (CG view) [40]. Relative synonymous codon usage (RSCU) of 12 PCGs was calculated using Phylosuite v1.2.3 [41]. The skew values were calculated according to the following formulas: AT skew = (A − T)/(A + T) and GC skew = (G − C)/(G + C) [42]. We used DnaSP [43] to calculate Ka/Ks ratios for 12 PCGs in the Pharidae family. 

### 3.4. Phylogenetic Analysis

To investigate the phylogenetic relationship of *N. chinensis*., mitogenome sequences of 69 species in Imparidentia were downloaded from the NCBI (Table 1). Mitogenomes of *Mimachlamys nobilis* and *Azumapecten farreri* were used as outgroups. Since most bivalve species lack the gene *atp8*, the phylogenetic analysis was conducted using amino acid sequences of 12 PCGs. Statistics for basic characteristics and the extraction of sequences were executed using PhyloSuite v1.2.3 [41]. A total of 12 PCGs were aligned using MAFFT v7.490 [44]. Ambiguously aligned fragments of 12 alignments were removed in batches using Gblocks 0.91b [45]. Then, the aligned nucleotide sequences were concatenated. ModelFinder v2.2.0 [46] was used to select the best-fit partition model (edge-linked) using a BIC criterion. 

Phylogenies were deduced using Bayesian inference (BI) and maximum likelihood (ML) methods. Maximum likelihood phylogenies were inferred using IQ-TREE v2.2.0 [47] within the Edge-linked partition model for 10,000 ultrafast bootstraps. Bayesian Inference phylogenies were inferred using MrBayes v3.2.7a [48] under a partition model (2 parallel runs, 2,000,000 generations), in which the initial 25% of the sampled data were discarded as burn-in. When the value of the average standard deviation of split frequencies drops below 0.01, it serves as an indication that the BI run has converged. Phylogenetic trees were visualized and annotated using the Interactive Tree of Life (ITOL) (https://itol.embl.de/itol.cgi (accessed on 16 August 2023)) [49].

## 4. Conclusions

In this study, we sequenced the complete mitochondrial genome of *N. chinensis* from the genus *Novaculina*. Its mitogenome contains 12 PCGs (except *atp8*), 22 tRNAs, and 2 rRNAs. Compared to other marine species in the Pharidae family, it exhibits a high AT content, small mitogenome size, and all genes encoded on the heavy strand. *Novaculina chinensis* diverges from species found in the freshwater basins of South and Southeast Asia, mainly in terms of the siphon morphology. More mitochondrial genomes from the species of the Pharidae family are necessary to explain this phenomenon and understand the family’s evolutionary progress. These findings also aid in conserving *N. chinensis* germplasm resources and studying its biological characteristics, providing vital molecular information for researching razor clams, which possess significant ecological and economic importance.

## Figures and Tables

**Table 1:** Organization of the mitogenome of *N. chinensis*.

Gene	Strand	Position (Start–End)	Length(bp)	Intergenic_Spacer	Start_Codon	Stop_Codon
*trnK-aaa*	+	1–67	67	-	-	-
*cox2*	+	113–922	810	45	ATG	TAG
*trnY-tac*	+	1086–1149	64	163	-	-
*nad4l*	+	1157–1444	288	7	ATG	TAA
*trnG-gga*	+	1446–1511	66	1	-	-
*trnP-cca*	+	1518–1582	65	6	-	-
*nad4*	+	1696–3057	1362	113	ATT	TAG
*trnH-cac*	+	3060–3123	64	2	-	-
*trnW-tga*	+	3126–3192	67	2	-	-
*trnR-cga*	+	3196–3260	65	3	-	-
*trnE-gaa*	+	3279–3345	67	18	-	-
*trnS2-tca*	+	3340–3402	63	−6	-	-
*nad3*	+	3403–3768	366	0	ATG	TAG
*trnT-aca*	+	3768–3833	66	−1	-	-
*trnI-atc*	+	3837–3902	66	3	-	-
*trnD-gac*	+	3918–3983	66	15	-	-
*trnQ-caa*	+	3984–4051	68	0	-	-
*trnC-tgc*	+	4062–4126	65	10	-	-
*trnA-gca*	+	4129–4193	65	2	-	-
*trnF-ttc*	+	4199–4262	64	5	-	-
*cox1*	+	4421–6100	1680	158	ATT	TAA
*trnL2-tta*	+	6128–6192	65	27	-	-
*nad1*	+	6193–7119	927	0	ATG	TAA
*trnL1-cta*	+	7124–7191	68	4	-	-
*trnV-gta*	+	7192–7255	64	0	-	-
*trnN-aac*	+	7256–7321	66	0	-	-
*nad5*	+	7322–9082	1761	0	ATT	TAA
*cob*	+	9119–10264	1146	36	ATG	TAA
*nad6*	+	10277–10807	531	12	ATG	TAG
*rrnL*	+	10808–12039	1232	0	-	-
*atp6*	+	12040–12738	699	0	ATG	TAA
*trnM-atg*	+	12751–12816	66	12	-	-
*rrnS*	+	12894–13742	849	77	-	-
*cox3*	+	13741–14529	789	−2	ATG	TAG
*trnS1-aga*	+	14529–14595	67	−1	-	-
*nad2*	+	14596–15648	1053	0	ATG	TAA

**Table 2:** The nucleotide composition and skewness of the mitogenome of *N. chinensis*.

Region	Size (bp)	A%	T%	G%	C%	AT%	GC%	AT-Skew	GC-Skew
Mitogenome	16,262	28.15	43.45	18.86	9.54	71.60	28.40	−0.214	0.328
PCGs	11,502	25.38	46.38	18.81	9.42	71.76	28.24	−0.293	0.333
tRNAs	1444	32.06	36.22	18.91	12.81	68.28	31.72	−0.061	0.192
rRNAs	2212	33.95	36.53	18.63	10.90	70.48	29.52	−0.04	0.26

**Table 3:** Codon number and RSCU of 12 PCGs in the mitogenome of *N. chinensis*. The asterisk (*) in the table indicates the stop codon.

Codon	Count	RSCU	Codon	Count	RSCU	Codon	Count	RSCU	Codon	Count	RSCU
UUU (F)	401	1.94	UCU (S)	146	2.89	UAU (Y)	148	1.91	UGU (C)	73	1.78
UUC (F)	12	0.06	UCC (S)	2	0.04	UAC (Y)	7	0.09	UGC (C)	9	0.22
UUA (L)	360	4.08	UCA (S)	46	0.91	UAA (*)	7	1.17	UGA (W)	58	1.2
UUG (L)	85	0.96	UCG (S)	9	0.18	UAG (*)	5	0.83	UGG (W)	39	0.8
CUU (L)	71	0.8	CCU (P)	90	3	CAU (H)	68	1.92	CGU (R)	32	1.94
CUC (L)	1	0.01	CCC (P)	3	0.1	CAC (H)	3	0.08	CGC (R)	2	0.12
CUA (L)	12	0.14	CCA (P)	24	0.8	CAA (Q)	33	1.43	CGA (R)	22	1.33
CUG (L)	1	0.01	CCG (P)	3	0.1	CAG (Q)	13	0.57	CGG (R)	10	0.61
AUU (I)	255	1.98	ACU (T)	80	2.52	AAU (N)	96	1.96	AGU (S)	56	1.11
AUC (I)	3	0.02	ACC (T)	2	0.06	AAC (N)	2	0.04	AGC (S)	5	0.1
AUA (M)	161	1.56	ACA (T)	39	1.23	AAA (K)	73	1.47	AGA (S)	110	2.18
AUG (M)	46	0.44	ACG (T)	6	0.19	AAG (K)	26	0.53	AGG (S)	30	0.59
GUU (V)	223	2.56	GCU (A)	141	3.34	GAU (D)	91	1.96	GGU (G)	165	2.06
GUC (V)	2	0.02	GCC (A)	2	0.05	GAC (D)	2	0.04	GGC (G)	11	0.14
GUA (V)	107	1.23	GCA (A)	24	0.57	GAA (E)	57	1.28	GGA (G)	88	1.1
GUG (V)	16	0.18	GCG (A)	2	0.05	GAG (E)	32	0.72	GGG (G)	56	0.7

## Data Availability

The sequenced mitogenome in this study has been deposited in GenBank (NC_077598.1).

## Supplementary Materials

The supporting information can be downloaded at: https://www.mdpi.com/article/10.3390/ijms25010067/s1.
