# Supplementary material for: The Complete Mitochondrial Genome and Phylogenetic Analysis of the Freshwater Shellfish Novaculina chinensis (Bivalvia: Pharidae)
Source: Int J Mol Sci. 2023 Dec 20;25(1):67. doi: 10.3390/ijms25010067 (PMC10778892; doi:10.3390/ijms25010067)
Supplement: Supplementary file 1 [file ijms-25-00067-s001.zip › ijms-2747339-supplementary.pdf]

Table S1. List of mitogenomes used in this study.

ID	Organism	Order	Superfamily	Family	Length	AT%
NC_025636.1	*Panopea globosa*	Adapedonta	Hiatelloidea	Hiatellidae	15469	63.7
NC_033538.1	*Panopea abrupta*	Adapedonta	Hiatelloidea	Hiatellidae	15381	64.4
NC_025635.1	*Panopea generosa*	Adapedonta	Hiatelloidea	Hiatellidae	15585	63.7
DQ632742.1	*Hiatella arctica*	Adapedonta	Hiatelloidea	Hiatellidae	18244	66.4
NC_016665.1	*Solen grandis*	Adapedonta	Solenoidea	Solenidae	16784	64.8
NC_077598.1	*Novaculina chinensis*	Adapedonta	Solenoidea	Pharidae	16262	71.6
NC_017616.1	*Solen strictus*	Adapedonta	Solenoidea	Solenidae	16535	62.6
MW727513.1	*Ensis leei*	Adapedonta	Solenoidea	Pharidae	16926	65.5
NC_011075.1	*Sinonovacula constricta*	Adapedonta	Solenoidea	Pharidae	17225	67
MT375556.1	*Siliqua minima*	Adapedonta	Solenoidea	Pharidae	17064	66.4
MW653805.1	*Cultellus attenuatus*	Adapedonta	Solenoidea	Pharidae	16888	66.4
NC_022194.1	*Fulvia mutica*	Cardiida	Cardioidea	Cardiidae	19110	64.5
NC_026558.1	*Tridacna squamosa*	Cardiida	Cardioidea	Cardiidae	20930	62.4
NC_035728.1	*Cerastoderma edule*	Cardiida	Cardioidea	Cardiidae	14947	58.2
NC_039945.1	*Tridacna derasa*	Cardiida	Cardioidea	Cardiidae	20760	65.3
NC_008452.1	*Acanthocardia tuberculata*	Cardiida	Cardioidea	Cardiidae	16104	60
MK249738.1	*Tridacna crocea*	Cardiida	Cardioidea	Cardiidae	19157	61.9
MT755624.1	*Tridacna noae*	Cardiida	Cardioidea	Cardiidae	20548	61.8
MG722975.1	*Hippopus hippopus*	Cardiida	Cardioidea	Cardiidae	22463	60.1
NC_050683.1	*Tridacna gigas*	Cardiida	Cardioidea	Cardiidae	19558	57.7
NC_046518.1	*Scrobicularia plana*	Cardiida	Tellinoidea	Scrobiculariidae	16170	66.6
NC_046519.1	*Limecola balthica*	Cardiida	Tellinoidea	Tellinidae	17492	63.6
NC_018374.1	*Semele scabra*	Cardiida	Tellinoidea	Semelidae	17117	59.2
NC_042422.1	*Gari elongata*	Cardiida	Tellinoidea	Psammobiidae	16766	62.2
NC_018373.1	*Nuttallia olivacea*	Cardiida	Tellinoidea	Psammobiidae	18182	65.3
NC_035987.1	*Donax vittatus*	Cardiida	Tellinoidea	Donacidae	17070	63.5
NC_035986.1	*Donax variegatus*	Cardiida	Tellinoidea	Donacidae	17195	60.4
NC_018372.1	*Hiatula diphos*	Cardiida	Tellinoidea	Psammobiidae	16352	63.3
NC_042423.1	*Sanguinolaria ovalis*	Cardiida	Tellinoidea	Psammobiidae	16460	61
NC_018371.1	*Iridona iridescens*	Cardiida	Tellinoidea	Tellinidae	16799	65.8
NC_042420.1	*Hiatula chinensis*	Cardiida	Tellinoidea	Psammobiidae	16333	61.3
NC_035985.1	*Donax trunculus*	Cardiida	Tellinoidea	Donacidae	17365	58.9
NC_035984.1	*Donax semiestriatus*	Cardiida	Tellinoidea	Donacidae	17044	61.9
NC_042421.1	*Hiatula acuta*	Cardiida	Tellinoidea	Psammobiidae	16352	63.3
NC_018376.1	*Solecurtus divaricatus*	Cardiida	Tellinoidea	Solecurtidae	16749	60.1
NC_013271.1	*Loripes lacteus*	Lucinida	Lucinoidea	Lucinidae	17321	62.1
NC_013275.1	*Lucinella divaricata*	Lucinida	Lucinoidea	Lucinidae	18940	63.7

NC_024738.1	*Mya arenaria*	Myida	Myoidea	Myidae	17947	65.7
NC_022709.1	*Arctica islandica*	Venerida	Arcticoidea	Arcticidae	18289	69.3
NC_046410.1	*Corbicula fluminea*	Venerida	Cyrenoidea	Cyrenidae	17423	70.5
NC_050989.1	*Villorita cyprinoides*	Venerida	Cyrenoidea	Cyrenidae	15880	68
MF981084.1	*Pliocardia ponderosa*	Venerida	Glossoidea	Vesicomyidae	16275	67.7
NC_028724.1	*Calyptogena magnifica*	Venerida	Glossoidea	Vesicomyidae	19738	68.4
MF981085.1	*Calyptogena extenta*	Venerida	Glossoidea	Vesicomyidae	16106	65.5
AP014557.1	*Abyssogena phaseoliformis*	Venerida	Glossoidea	Vesicomyidae	19424	70.4
NC_044766.1	*Archivesica marissinica*	Venerida	Glossoidea	Vesicomyidae	17374	65.4
MG431821.1	*Pseudocardium sachalinense*	Venerida	Mactroidea	Mactridae	17978	59.4
NC_023384.1	*Lutraria rhynchaena*	Venerida	Mactroidea	Mactridae	16927	62.3
NC_025510.1	*Mactra chinensis*	Venerida	Mactroidea	Mactridae	17285	63.7
NC_021375.1	*Mactra antiquata*	Venerida	Mactroidea	Mactridae	17199	64.2
NC_036766.1	*Lutraria maxima*	Venerida	Mactroidea	Mactridae	17082	63.9
NC_026728.1	*Saxidomus purpurata*	Venerida	Veneroidea	Veneridae	19637	66.2
NC_038063.1	*Dosinia japonica*	Venerida	Veneroidea	Veneridae	17693	70
NC_016890.1	*Paratapes textilis*	Venerida	Veneroidea	Veneridae	18561	64.3
NC_016891.1	*Paratapes undulatus*	Venerida	Veneroidea	Veneridae	18154	64.9
NC_045888.1	*Macridiscus multifarius*	Venerida	Veneroidea	Veneridae	20171	67.6
NC_014809.1	*Meretrix lusoria*	Venerida	Veneroidea	Veneridae	20268	67.9
NC_045870.1	*Macridiscus melanaegis*	Venerida	Veneroidea	Veneridae	20738	67.1
NC_048487.1	*Mercenaria mercenaria*	Venerida	Veneroidea	Veneridae	18365	69
NC_037916.1	*Dosinia altior*	Venerida	Veneroidea	Veneridae	17536	69.6
NC_016174.1	*Meretrix lamarckii*	Venerida	Veneroidea	Veneridae	21209	65.7
KU097333.1	*Cyclina sinensis*	Venerida	Veneroidea	Veneridae	21799	73
NC_012767.1	*Meretrix petechialis*	Venerida	Veneroidea	Veneridae	19567	68.3
NC_037917.1	*Dosinia troscheli*	Venerida	Veneroidea	Veneridae	17229	69.7
NC_014579.1	*Paphia euglypta*	Venerida	Veneroidea	Veneridae	18643	66.9
NC_035757.1	*Ruditapes decussatus*	Venerida	Veneroidea	Veneridae	18995	63
NC_013188.1	*Meretrix meretrix*	Venerida	Veneroidea	Veneridae	19826	68.4
NC_022924.1	*Meretrix lyrata*	Venerida	Veneroidea	Veneridae	21625	70.5
NC_016889.1	*Paphia amabilis*	Venerida	Veneroidea	Veneridae	19629	63.3

Table S2. The Ka/Ks ratio for 12 protein coding genes of Pharidae bivalves.

Gene	Ka/Ks Ratio
atp6	0.093720433
cox1	0.063960132
cox2	0.10541316
cox3	0.071789364
cytb	0.106053968
nad1	0.102494324
nad2	0.156146896
nad3	0.10770631
nad4	0.142094014
nad4L	0.131802675
nad5	0.155082086
nad6	0.231071968
